# Influence of environmental factors on the genetic variation of the aquatic macrophyte *Ranunculus subrigidus* on the Qinghai-Tibetan Plateau

**DOI:** 10.1186/s12862-019-1559-0

**Published:** 2019-12-19

**Authors:** Zhigang Wu, Xinwei Xu, Juan Zhang, Gerhard Wiegleb, Hongwei Hou

**Affiliations:** 1 Institute of Hydrobiology, Chinese Academy of Science, Wuhan, China; 2 College of Life Science, Wuhan University, Wuhan, China; 3 Department of Ecology, Faculty of Environment and Natural Sciences, Brandenburg University of Technology Cottbus-Senftenberg, Cottbus, Germany

**Keywords:** Alpine wetland, Aquatic plant, Genetic diversity, Isolation-by-environment, *Ranunculus* section *Batrachium*

## Abstract

**Background:**

Due to the environmental heterogeneity along elevation gradients, alpine ecosystems are ideal study objects for investigating how ecological variables shape the genetic patterns of natural species. The highest region in the world, the Qinghai-Tibetan Plateau, is a hotspot for the studies of evolutionary processes in plants. Many large rivers spring from the plateau, providing abundant habitats for aquatic and amphibious organisms. In the present study, we examined the genetic diversity of 13 *Ranunculus subrigidus* populations distributed throughout the plateau in order to elucidate the relative contribution of geographic distance and environmental dissimilarity to the spatial genetic pattern.

**Results:**

A relatively low level of genetic diversity within populations was found. No spatial genetic structure was suggested by the analyses of molecular variance, Bayesian clustering analysis and Mantel tests. Partial Mantel tests and multiple matrix regression analysis showed a significant influence of the environment on the genetic divergence of the species. Both climatic and water quality variables contribute to the habitat heterogeneity of *R. subrigidus* populations.

**Conclusions:**

Our results suggest that historical processes involving long-distance dispersal and local adaptation may account for the genetic patterns of *R. subrigidus* and current environmental factors play an important role in the genetic differentiation and local adaptation of aquatic plants in alpine landscapes.

## Background

By limiting gene flow within species, both spatial distance and environmental heterogeneity influence evolutionary as well as ecological processes [1, 2]. Gene flow among populations may be reduced due to the geographic isolation through the combined force of dispersal limitation and genetic drift [3]. A linear relationship between genetic differentiation and geographic distance (isolation-by-distance, IBD) indicates a general tendency for speciation or intraspecific differentiation [3]. The pattern will be disrupted when migration, colonization or mating are under the influence of ecological factors [1, 4, 5]. Recently, genetic isolation-by-environment (IBE) was discovered as another important driver of adaptive divergence processes, related to both small-scale heterogeneity and short-term changes of the environment [6, 7].

In alpine ecosystem, physical barriers and a complex topography can significantly reduce the dispersal of organisms [8, 9]. On the other hand, elevation gradients in alpine regions lead to increased habitat heterogeneity as to photoperiod, radiation, soil characteristics or biotic factors in addition to the climatic gradient [10]. Thus, plant communities in mountains provide an important context for studying mechanisms of genetic structuring related to patterns of genetic isolation among populations at different scales. Manel et al. [11] have shown that alpine environments may facilitate rapid adaptation in plants. Several subsequent studies supported these findings [12, 13]. However, more studies on representative taxa are necessary for a comprehensive understanding of the issue [2, 14, 15]. The highest region in the world, the Qinghai-Tibetan Plateau (QTP), is highly vulnerable to global climate change [16, 17]. It is characterized by wide variations in atmospheric pressure, solar radiation, topographies, wind regimes and mesoclimates [18, 19]. The plateau is considered to be an ideal ‘natural laboratory’ for investigations on adaptive evolution [20–22].

The QTP gives rise to many large rivers of eastern and southern Asia. Therefore, river channels, lakes and other wetlands are found in great abundance [23]. The QTP provides a great wealth of habitats for vascular aquatic plants [24], an important functional group in freshwater [25, 26]. Aquatic plants show a limited taxonomic differentiation and low intraspecific genetic variation, due to the broad tolerance ranges and plasticity of genotypes, as well as preferential clonal reproduction [27]. The way aquatic plants react to the heterogeneous and harsh environments at multiple scales is poorly understood [28, 29]. Phylogeographic studies suggest an early natural establishment of aquatic plants on the QTP in mid-Pleistocene [30, 31]. The complexity of environments may have promoted specialization and divergence of aquatic plants [32]. To date, landscape genetic studies on aquatic plants of the QTP are rare. A previous study showed that geographic isolation could be a main factor influencing the spatial genetic structure of *Myriophyllum spicatum* L. on southeastern edge of the QTP [33].

*Ranunculus* section *Batrachium* (Ranunculaceae) comprises about 30 species with aquatic or semiaquatic habitat requirements [34, 35]. *Ranunculus* sect. *Batrachium* species show worldwide distributions from arctic to meridional zones. They are characterized by extreme phenotypic plasticity, diverse breeding systems, polyploidization, and network evolution by means of frequent hybridization [34, 36–38]. *Ranunculus* sect. *Batrachium* species are frequently found across the QTP [24]. We regard them as representative taxa to understand how the environment is influencing genetic variation of aquatic plants at a large geographic scale. *Ranunculus* sect. *Batrachium* on the QTP comprises three distinct species (*Ranunculus subrigidus* W. B. Drew, *Ranunculus flavidus* (Hand.-Mazz.) C.D.K. Cook and *Ranunculus trichophyllus* Chaix) based on our field identifications and herbarium studies. The species differ in flower characteristics, life cycle and genetics [35, 39]. In present study, our aim was estimating the extent and spatial pattern of genetic variation of *R. subrigidus*. The species has an Amphipacific distribution [35]. *R. subrigidus* tends to colonize hard water habitats, sometimes even brackish waters [35], which makes the species one of most common aquatic species on the QTP. Absence of IBD pattern has been revealed in *R.* sect. *Batrachium* on the QTP, whereas the effects of environment were not concerned [30, 40]. Severely diverse habitats might promote the divergence in aquatic plants [41–43]. We therefore conducted further landscape genetic analysis to evaluate the hypothesis that the environmental variables on the QTP might influence the population genetic structure of *R. subrigidus*.

## Results

A total of 164 genets were identified in the 13 populations of *R. subrigidus.* The number of multilocus genotypes per population ranged from 5 to 20, all ramets of MQ, NM and CM were from different genets (Table 1). The number of alleles per locus (NA), the expected heterozygosity (H_E_) and the observed heterozygosity (H_O_) ranged from 1.294 to 2.471, from 0.035 to 0.122 and from 0.078 to 0.24, respectively (Table 1).

**Table 1:** Geographic origins, sample sizes (N), genetic diversity and effective population size (*θ*, with 95% confidence intervals) for the 13 *Ranunculus subrigidus* populations on the Qinghai-Tibetan Plateau

Code	Location	Coord.	Alt.	N	G	NA	H_E_	H_O_	*θ*
South and West QTP (SWQTP)									
GE1	Geer, Tibet	31.89°N 80.16°E	4387	21	17	1.529	0.098	0.097	3.276 (0–6.9)
GE2	Geer, Tibet	32.40°N 80.83°E	4524	14	10	1.529	0.122	0.153	5.616 (0–7.8)
GJ	Geji, Tibet	32.10°N 81.79°E	4616	14	12	1.588	0.08	0.131	4.377 (0–6.6)
ZB	Zhongba, Tibet	29.70°N 84.13°E	4569	19	14	1.588	0.17	0.158	3.177 (0–6.9)
SG	Saga, Tibet	29.42°N 85.24°E	4701	20	7	1.412	0.035	0.078	4.379 (0–7.9)
DR	Dingri, Tibet	28.59°N 86.83°E	4314	20	10	1.294	0.106	0.094	4.561 (0–8.1)
NM	Nanmulin, Tibet	30.00°N 89.10°E	4311	19	19	2.059	0.121	0.24	3.674 (0–7.3)
CM	Cuomei, Tibet	28.77°N 91.67°E	4626	20	20	2.471	0.079	0.23	5.040 (0–7.7)
Northeast QTP (NEQTP)									
QM	Qumalai, Qinghai	35.15°N 93.04°E	4704	16	8	1.412	0.088	0.097	15.966 (2.5–22.4)
DL	Delinha, Qinghai	37.25°N 97.03°E	2816	13	5	1.294	0.081	0.093	4.400 (0–7.9)
MD	Maduo, Qinghai	34.86°N 97.49°E	4274	22	21	2.353	0.104	0.2	13.568 (0.5–17.2)
MQ	Maqin, Qinghai	33.91°N 99.56°E	4036	15	15	1.941	0.067	0.132	4.384 (0–8)
DT	Xining, Qinghai	37.10°N 101.57°E	2628	15	6	1.412	0.086	0.098	5.166 (0.1–8.5)
Average				17.5	12.6	1.683	0.095	0.139	

*G* Number of genets, *NA* Number of alleles per locus, *H*_*E*_ Expected heterozygosity, *H*_*O*_ Observed heterozygosity

**Fig. 1:**
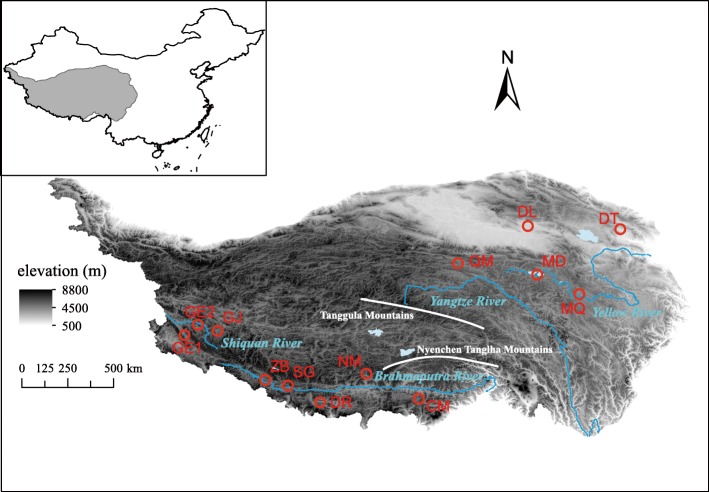
The sampling sites of *Ranunculus subrigidus* on the Qinghai-Tibetan Plateau are mapped with relative code using ArcGIS. The elevation range, main rivers, lakes and mountains are visualized, and the sources are supported by National Earth System Science Data Center, National Science & Technology Infrastructure of China (http://www.geodata.cn)

**Fig. 2:**
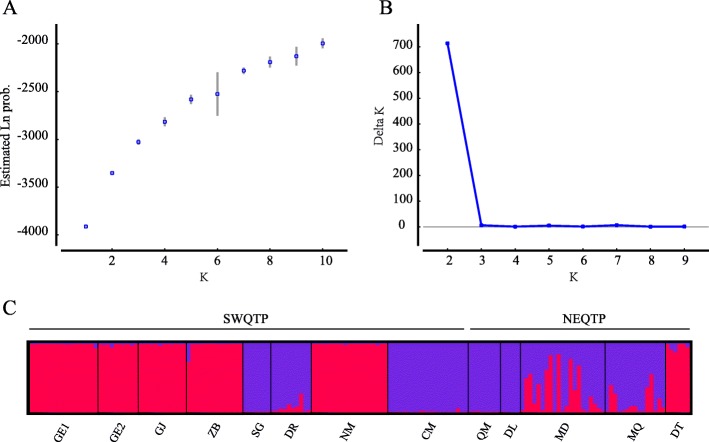
Modelling of the number of genetic clusters in *Ranunculus subrigidus* based on the Estimated Ln Prob. (**a**) and ΔK (**b**) in STRUCTURE. (**c**) The bar plot depicts the genetic assignment of *Ranunculus subrigidus* when K = 2, a single vertical bar displays the membership coefficient of each genet. The codes of sample site are labeled

An analysis of molecular variance (AMOVA) suggested only 4% of genetic variation was partitioned between subregions, and about half of the genetic variation was distributed among populations (Table 2). Two genetic clusters were suggested, inferred from STRUCTURE analysis (Fig. 2a and b). Cluster A consists of GE1, GE2, GJ, ZB, NM (the southern and western QTP, SWQTP) and DT (the northeast QTP, NEQTP), when Cluster B consists of SG, DR, CM (SWQTP) and QM, DL (NEQTP) (Fig. 2). Genetic admixture was found in a lake population MD and an adjacent downstream population MQ (Figs. 1 and 2c).

**Table 2:** Analysis of molecular variance of 13 populations of *Ranunculus subrigidus* on the Qinghai-Tibetan Plateau

Source of variation	D.F.	S.S.	E.V.	P.V.	*p*
Between subregions	1	58.519	0.107	4%	< 0.001
Among populations within subregions	11	398.199	1.397	48%	< 0.001
Within populations	315	435.014	1.406	49%	< 0.001
Total	327	891.732	2.911	100%	

*D.F*. Degrees of freedom, *S.S*. Sum of squares, *E.V*. Estimated variance, *P.V*. Percentage of variation

The pairwise genetic distance among populations was calculated on the individual and population level respectively. The results showed that the matrices of genetic distance estimated with different approaches were significantly correlated (*r =* 0.644, *p* < 0.001). The DT and DL, DT and SG, DL and CM were the most divergent populations suggested by both approaches (Additional file 1: Tables S1 & S2).

Multiple MIGRATE runs show consistent result for direct estimation of gene flow. Asymmetric migration rates (*M*) varied from 0.678 (DL - > NM) to 11.357 (QM - > SG) (Additional file 1: Table S3), suggesting limited migration between populations. Small effective population sizes (*θ* < 50) were also indicated by MIGRATE (Table 1). The number of migrants per generation (*N*m) was calculated using the *N*m = (*M* * *θ*)/4, the migrants between *R. subrigidus* populations on the QTP ranged from 0.623 to 25.506 per generation (Additional file 1: Table S3). Immigration to the lake population MD and high-elevation population QM was higher than to others (Additional file 1: Table S3). For the populations at the northeast edge of the QTP (DT and DL), gene flow to other populations was relatively low (Additional file 1: Table S3).

**Fig. 3:**
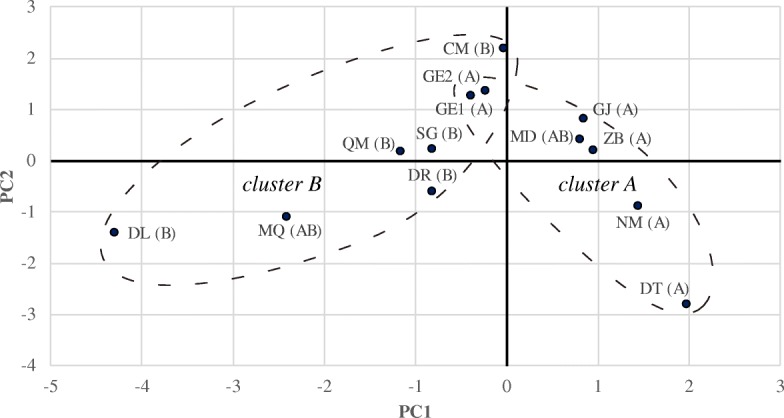
The environmental space of the 13 *Ranunculus subrigidus* populations quantified with PCA axes. Coordinates of the first two axes are presented. The genetic cluster of each population identified by STRUCTURE are labeled

The environmental space of 13 populations was quantified using principle component analysis (PCA) on environmental variables, including elevation, climate and water quality of the sample sites (Table 3, Fig. 3, Additional file 1: Table S4). The first two axes explain 62.5% of environmental variation. The first axis of the environmental space is closely associated with temperature seasonality, precipitation and water quality, and the second axis is closely associated with elevation and temperature (Table 3). The populations assigned to different genetic clusters occupied distinct environment space (Fig. 3). The environment dissimilarity among populations were calculated with Euclidean distance based on the coordinates of first two PC axes (the results of subsequent analyses were consistent when first three PC axes were used, not showed). We found no significant relationship between the genetic distance/migration rate and geographic distance within all genetic similarity matrices (Table 4). The relationship between genetic distance/gene flow rate and environmental dissimilarity was significant (population-based: *r =* 0.409, *p =* 0.023; individual-based: *r =* 0.496, *p =* 0.008; migration rate: *r* = 0.553, *p* = 0.001, Table 4). The relationship was maintained when we set geographic distance as a control (population-based: *r =* 0.351, *p =* 0.027; individual-based: *r =* 0.471, *p =* 0.011; migration rate: *r* = 0.542, *p* = 0.001, Table 4). The Multiple matrix regression with randomization (MMRR) analysis also showed a high relationship between ecology and genetic differentiation (population-based: β = 0.356, *p =* 0.031; individual-based: β = 0.489, *p =* 0.019; migration rate: β = 0.568, *p =* 0.001, Table 5), whereas the effects of geographic isolation were not significant (population-based: β = 0.167, *p =* 0.173; individual-based: β = 0.023, *p =* 0.795; migration rate: β = 0.046, *p =* 0.616, Table 5). Distance-based redundancy analysis (dbRDA) revealed the first two principal component variables could explain one-third of the variations in genetic differentiation, and 58.7% of the variations in migration rates between populations (Additional file 1: Tables S5). Significant effect of PC1 on the genetic pattern was suggested by regressions on different genetic similarity matrices (Additional file 1: Tables S5).

**Table 3:** Relative contribution of environmental variable to the first two axes of the PCA analyses

Environmental variable		PC1 (39.0%)	PC2 (23.5%)
Climate	altitude	0.129	0.615
	GST	0.173	0.627
	ELT	−0.167	0.201
	TS	−0.459	0.014
	AP	−0.541	−0.141
Water quality	pH	−0.488	0.112
	salinity	0.430	−0.394

*GST* Growing season temperature, *ELT* Extreme low temperature, *TS* Temperature seasonality, *AP* Annual precipitation

**Table 4:** Simple and partial Mantel tests on the correlation between genetic similarity matrices (Rousset’s *â*, *F*_*ST*_ and *M*), geographic distance (Geo) and environmental dissimilarity (Env) of *Ranunculus subrigidus* populations on the Qinghai-Tibetan Plateau

Genetic distance Index	Landscape feature	Controlled	r	*p*
Rousset’s *â*	Geo		0.180	0.077
	Env		0.496	0.008
	Geo	Eco	0.025	0.389
	Env	Geo	0.471	0.011
*F*_*ST*_	Geo		0.201	0.060
	Env		0.409	0.021
	Geo	Eco	0.138	0.156
	Env	Geo	0.351	0.027
*M*	Geo		0.136	0.108
	Env		0.553	0.001
	Geo	Eco	0.052	0.707
	Env	Geo	0.542	0.001

**Table 5:** Result of MMRR analysis on the correlation between genetic similarity matrices (Rousset’s *â*, *F*_*ST*_ and *M*), geographic distance (Geo) and environmental dissimilarity (Env) of *Ranunculus subrigidus* populations on the Qinghai-Tibetan Plateau

Genetic distance Index	Landscape feature	β	*p*
Rousset’s *â*	Geo	0.022	0.795
	Env	0.489	0.019
*F*_*ST*_	Geo	0.167	0.173
	Env	0.356	0.031
*M*	Geo	0.046	0.616
	Env	0.568	0.001

## Discussion

In the present study, low genetic variation was revealed in *R. subrigidus* on the QTP with polymorphic microsatellite markers (Table 1). Most ramets present distinct multilocus genotypes, implying the clonal reproduction was not predominant in the species (Table 1). The value of Ho (0.139, in average) and *F*_*ST*_ (0.508, globally) in *R. subrigidus* resembled a species with a selfing breeding system (H_O_ = 0.05, *F*_*ST*_ = 0.42, [44]). The mean H_E_ (0.095) is much lower than that of a species with a regional distribution [44]. A lack of genetic variation has been revealed in population genetics studies on *R*. sect. *Batrachium* by ISSR and cpDNA markers [45, 46]. It has also been found for Russian populations of *R. subrigidus* [47]. Self-compatibility and vegetative reproduction are common in *R*. sect. *Batrachium* species, which may influence the level of genetic variation in different habitats [48–50]. The small population and effective population size in *R. subrigidus* caused by severe isolation and aquatic habitat fragmentation on the QTP make the plants more vulnerable to inbreeding, genetic drift/founder effects and ecological selection (Table 1) [24, 51], all of which mainly influence the level of genetic variation in *R. subrigidus*.

Absence of spatial genetic structure in *R. subrigidus* on the QTP is indicated by the following results. The genetic clusters identified in the present study are not corresponding to the two distant subregions, as suggested by STURCTURE (Fig. 2). The hierarchical analyses showed a significant genetic variation component among and within populations, but no genetic differentiation between the subregions (Table 2). No significant linear relationship between genetic differentiation and geographic distance was found (Table 4). IBD is regarded as the general pattern of neutral genetic differentiation in plants [1–3], especially in alpine landscapes due to the greater impact of historical processes such as glaciation and post-glaciation colonization [52]. Most available studies on aquatic plants also showed that geographic isolation played an essential role on their evolutionary processes [27, 53]. Nevertheless, the current genetic structure of alpine plants could be shaped by repeated range changes. It more likely occurs in some cold tolerant taxa, which might have survived the glaciation in situ on the plateau platform [20, 54]. According to previous studies on phylogeography, *R. subrigidus* might have experienced expansions and retreats in mid-to-late Pleistocene [30], undergoing a similar evolutionary history as the aquatic herb *Hippuris vulgaris* L. [31]. On the other hand, compared to the terrestrial plants on the QTP, the dispersal of aquatic plants was facilitated by rivers or streams, while dispersal by waterfowl may have allowed crossing watersheds and physical barriers such as high mountains [55–57]. The dispersal of propagules in *Ranunculus* species through duck guts has been proved [56, 58]. The potential of long distance dispersal in *R. subrigidus* contributes to the genetic similarity between distant populations. Previous studies suggested the important role of passive dispersal on the genetic structuring in aquatic plants in different scales [59–61]. MD (Zhaling-Eling Lake) is one of largest lakes for the breeding of migration waterfowls on the QTP [62]. We therefore consider the random movements of *R. subrigidus* via transport by waterfowl or humans might be a viable explanation for the current distribution (Fig. 2). The assumption is supported by the relatively high gene flow rates between MD and some distant populations (e.g. GE1, GJ) (Additional file 1: Table S3). More empirical studies are necessary on how waterfowl or human activities contribute to the dispersal and divergence of aquatic plant populations [63].

IBE may be another important cause for the absence of IBD, when the influence of the contemporary environmental conditions is strong [2, 7]. In *R. subrigidus*, IBE was confirmed by both partial Mantel test and multiple regression under the effect of geography as a covariate (Tables 4 and 5). We also found that the two genetic clusters had different niche requirements (Fig. 3). It implies that the effective dispersal among *R. subrigidus* populations on the QTP was significantly influenced by environmental factors. For instance, near-by populations in significantly different environments (e.g. DT and DL, Figs. 1 and 3) were highly divergent despite relatively low rates of movement (Additional file 1: Tables S1, S2 and S3). Gene flow among adjacent populations also benefited from environmental similarity (e.g. SG/DR vs ZB/NM, Fig. 1, Additional file 1: Tables S1, S2 and S3). Hostile environments on the QTP, especially short growing seasons and low temperature, negatively influence both photosynthetic rates and reproduction of plants [18, 64]. It would limit the gene flow when natural selection operates on the immigrates or their offspring with local individuals [4, 7, 65]. Climate was considered as one of the best predictors for the differentiation among alpine populations [22, 66]. It was identified as the primary driver for the IBE pattern in landscape genetic studies [67, 68]. Temperature, precipitation and water quality mainly contributed to environmental heterogeneity of *R. subrigidus* (Table 3), suggesting that they might induce the differentiation and potential adaptation of *R. subrigidus* populations. The results of multivariate regression also support these variables significantly influence the genetic pattern of *R. subrigidus* (Additional file 1: Table S5). Temperature seasonality greatly varied among the sites of *R. subrigidus* on the QTP, and significantly differed between the two genetic clusters (Additional file 1: Table S4). The factor is generally known to shaping life history strategies [69]. It may elicit adaptive traits on the growth and reproduction of plants [69], which could promote the divergence of the populations from different seasonal environments. The precipitation regime might decide the geographic pattern and adaptive strategies of aquatic plants as well [70]. On the QTP, precipitation is significantly reduced with the increasing altitude. Insufficient precipitation might promote the plants to be tolerant to droughts and competitive at low water levels [71]. It also affects habitat quality such as the mineralization (water salinity, pH) of isolated lakes [71, 72]. The habitat specificity could shape the spatial pattern of species richness and community [43] and enhance intraspecies genetic differentiation of aquatic macrophytes [73, 74], due to the combined effect of water qualities. These variables might simultaneously drive the local adaptation of *R. subrigidus* in alpine environments. Although it was generally expected that local adaptation served as a week barrier for gene flows in aquatic macrophytes due to the insignificance of sexual recombination [41], adaptations to broad climatic gradients or extreme water environments has been proved in some widespread species [75, 76]. We therefore assume that large-scaled environmental heterogeneities of climatic and habitat features might drive the local adaptation, simultaneously maintaining the genetic divergence of *R. subrigidus* on the QTP.

## Conclusions

*R. subrigidus* is a representative taxon to study the influence of alpine environments on the genetic structuring of wetland plants. Similar as in many other species of *R*. sect. *Batrachium*, a lack of genetic variation was revealed in *R. subrigidus*. Severe isolation and habitat fragmentation on the QTP make the wetland plants more vulnerable to genetic drift, inbreeding and selection caused by heterogeneous and harsh environments. An IBE pattern, but no IBD pattern was found in *R. subrigidus*, implying a significant influence of the environment (especially climatic seasonality) on the genetic divergence within the species. Long-distance dispersals and historical processes may also contribute to the patterns. The results would further our understanding about how aquatic plants react and adapt to the heterogeneous and harsh environments.

## Methods

### Field sampling

Field sampling was carried out in the August of 2013 and 2016, respectively. All samples were collected from small water bodies in wetlands and from lakeshores. Idenfication of specimens in the field followed [35]. Water quality (salinity and pH) were measured three times at different places of the sites for an average before sampling the plant material (Additional file 1: Table S4), using a handheld multiparameter meter (PROPLUS, YSI, USA). We sampled a total of 228 individuals at thirteen sites throughout the QTP. Size ranged from 13 to 22 individuals per site (Table 1). As *R. subrigidus* can extend clonally via rhizomes, a genet (genetic individual) may consist of individual shoots (ramets) covering several m^2^. All samples were therefore collected at an interval of at least 20 m. Fresh leaves were dried in silica gel in the field and frozen at − 20 °C in the laboratory.

### Clone identification and genetic diversity

Genomic DNA was extracted using the Novel DNA Plant Kit (Kangwei Biotech, Beijing, China). Thirteen self-developed EST-SSRs and four feasible SSRs isolated in *Ranunculus* species were used in *R. subrigidus* [39]. All PCR amplifications were performed in 20 μL reaction mixtures containing 1.5 μL genomic DNA (~ 30 ng/μL), 0.5 μL of each primer (10 μM), and 10 μL 1x master PCR Mix (Tiangen Biotech, Beijing, China). The PCR conditions comprised an 5 min initial denaturation step at 95 °C; followed by 35 cycles of 30 s at 95 °C, 30 s at suggested annealing temperature according to Wu et al. [39], 1 min at 72 °C; and a final extension at 72 °C for 7 min. PCR products were analyzed on the ABI 3730XL analyzer (Applied Biosystems, Foster City, CA, USA). The microsatellite genotyping was performed using the GeneMarker V.1.5 software (SoftGenetics, State College, Pennsylvania, PA, USA).

As the sampling distance of 20 m does not guarantee that the samples do not belong to the same genet (e.g. in case of short-distance propagation by fragments), we carried out an analysis of clone identification using RClone [77] in R 3.3.3 (R Development Core Team 2017). The probability-based method can identify identical multilocus genotypes (MLGs) having arisen via sexual reproduction (*p*_sex_) [78]. Duplicate MLGs were removed for the following analyses. Number of alleles and observed/expected heterozygosity were estimated to evaluate the genetic diversity of each population using GenALEx 6.501 [79].

### Genetic structure and gene flow

*Ranunculus subrigidus* populations on the QTP were divided into two geographic subregions: the southern and western QTP (SWQTP, comprising both Shiquan River and Brahmaputra River basins) and the northeast QTP (NEQTP, comprising the upper reaches of Yangtze River and Yellow River basins). The two subregions are separated by the great mountains in the central QTP such as Tanggula Mountains and Nyenchen Tanglha Mountains (Fig. 1). We calculated the distribution of genetic variation between subregions, and among and within populations based on an AMOVA using Arlequin ver. 3.5 [80]. A Bayesian clustering method approach was also used for genetic cluster assignment for individuals, implemented in STRUCTURE 2.3.4 [81, 82]. Twenty independent runs were performed for each K value (K = 1 to 10) with a burn-in period of 200,000 iterations and 1,000,000 MCMC iterations under the admixture model. The best-fit number of clusters was determined using STRUCTUREHARVEST [83]. Visualization of the Bayesian clustering was conducted in DISTRUCT 1.3 [84].

The genetic distance was measured based on the individual and population level respectively. Genetic relatedness between individuals was presented by Rousset’s *â* [85], using SPAGeDi 1.3 [86]. The index was suggested to be one of the most accurate individual-based genetic distance metrics for landscape genetic analysis [87]. Considering the individuals from the same site can not represent the populations independently, we randomly picked one genet per sampling site to calculate pairwise relatedness and repeated the calculations 100 times. The averaged value of genetic distance for each pair of localities was subsequently obtained. The pairwise inter-population *F*_*ST*_ was also calculated using Genelax 6.501 [88]. Given the accuracy of genetic estimation could be influenced by the small sample size (the number of genets per site ≥5, see results), we gave 100 replicates of sub-population random sampling of five individuals from all sites and calculated the respective *F*_*ST*_ estimates [68]. We found genetic distance matrix across different replicates were highly correlated (Mantel *r* = 0.841 ± 0.065, all *P* values < 0.001), which indicated a genet number of no less than five can provide reliable estimation of pairwise *F*_*ST*_ values. The matrices of genetic distance measured with individual-based and population-based approach were used for the subsequent analysis respectively.

We used the program MIGRATE 3.7.2 [89] to calculate historical mutation-scaled asymmetric immigration rates (*M*) for direct measurement of gene flow. The effective population size (*θ*) was also generated in the program. The data were analyzed with Bayesian inference strategy and followed a Brownian motion model. The starting values of both *θ* and *M* were generated using *F*_*ST*_ calculation method. We specified uniform priors for *θ* with a minimum of 0, maximum of 200, and a delta of 20, and for M with a minimum of 0, maximum of 100, and a delta of 10. We set static heating with four chains (temperatures: 1, 1.5, 3.0, 100,000.0), a sampling increment of 100, 50,000 recorded steps, and a burn-in of 50,000.

The pattern of isolation by distance was tested by the Mantel tests with 10,000 permutations based on pairwise genetic similarity index and geographic distance among all populations**,** using the VEGAN package in R [90]**.**

### Landscape genetics

Growing season temperature, extreme low temperate, temperature seasonality and annual precipitation were introduced as climate variables (Table 3, Additional file 1: Table S4), which were derived from data collected at meteorological stations across China based on the geographic coordinates [91]. The environmental space of all sample sites was quantified and visualized using PCA, with standardized variables of the climate and water quality (pH and salinity) in R software. The environmental space was constructed by the first two PCA axes (Table 3). The principal component variables of the studied sites were used to calculate environmental (Euclidean) distances between populations.

All index of genetic distance and gene flow were suitable for the landscape genetic studies [67]. Because MIGRATE produced asymmetrical pairwise migration rates, we made averages on immigrates and emigrates to obtain single values between populations for subsequent analyses [92]. A combination of partial Mantel test and matrix regression analysis was employed to evaluate the independent contribution of environmental heterogeneity and geographic isolation to the genetic variation of *R. subrigidus* on the QTP. Partial Mantel tests with 10,000 permutations were performed between genetic distance and geographic/environmental distance under the influence of the other factor, using R package VEGAN. MMRR was implemented with 10,000 permutations in R with the MMRR function script [93]. We also used distance-based redundancy analysis (dbRDA) to determine the contributions of environmental principal component variables in driving the genetic patterns. The dbRDA performed a multivariate regression on the response variable (genetic distance/gene flow) using the VEGAN package.

## Supplementary information

**Additional file 1:** **Table S1** Pairwise individual-based genetic distance (Rousset’s *â*) between 13 *Ranunculus subrigidus* populations. **Table S2** Pairwise population-based genetic distance (*F*_*ST*_) between 13 *Ranunculus subrigidus* populations. **Table S3** Estimates of historical asymmetric migration rates (*M*)/ number of migrants per generation (*N*m) between populations of *Ranunculus subrigidus*. **Table S4** Measures of environmental variables of 13 *Ranunculus subrigidus* populations. **Table S5** Results of dbRDA on the contributions of environmental principal component variables on the genetic pattern.

## Data Availability

The data set supporting the results of this article is available in the Dryad repository, in 10.5061/dryad.j6q573n8v.

## Abbreviations

AMOVA: An analysis of molecular variance
dbRDA: distance-based redundancy analysis
HE: Expected heterozygosity
HO: Observed heterozygosity
IBD: Isolation-by-distance
IBE: Isolation-by-environment
MLGs: Multilocus genotypes
MMRR: Multiple matrix regression with randomization
NA: Number of alleles per locus
NEQTP: Northeast Qinghai-Tibetan Plateau
PCA: Principle component analysis
QTP: Qinghai-Tibetan Plateau
SWQTP: Southern and western Qinghai-Tibetan Plateau
