# Supplementary material for: Influence of environmental factors on the genetic variation of the aquatic macrophyte Ranunculus subrigidus on the Qinghai-Tibetan Plateau
Source: BMC Evol Biol. 2019 Dec 19;19:228. doi: 10.1186/s12862-019-1559-0 (PMC6921560; doi:10.1186/s12862-019-1559-0)
Supplement: Supplementary file 1 — Additional file 1: Table S1 Pairwise individual-based genetic distance (Rousset’s â) between 13 Ranunculus subrigidus populations. Table S2 Pairwise population-based genetic distance (FST) between 13 Ranunculus subrigidus populations. Table S3 Estimates of historical asymmetric migration rates (M)/ number of migrants per generation (Nm) between populations of Ranunculus subrigidus. Table S4 Measures of environmental variables of 13 Ranunculus subrigidus populations. Table S5 Results of dbRDA on the contributions of environmental principal component variables on the genetic pattern. [file 12862_2019_1559_MOESM1_ESM.docx]

**Table S1.** Pairwise individual-based genetic distance (Rousset’s *â*) between 13 *Ranunculus subrigidus* populations

|  | DT | DL | QM | MQ | MD | NM | DR | SG | ZB | GE1 | GE2 | GJ |
| --- | --- | --- | --- | --- | --- | --- | --- | --- | --- | --- | --- | --- |
| DL | 4.299 |  |  |  |  |  |  |  |  |  |  |  |
| QM | 2.910 | 2.447 |  |  |  |  |  |  |  |  |  |  |
| MQ | 1.956 | 3.331 | 1.911 |  |  |  |  |  |  |  |  |  |
| MD | 2.737 | 2.702 | 2.098 | 1.859 |  |  |  |  |  |  |  |  |
| NM | 2.428 | 4.430 | 3.152 | 2.844 | 3.044 |  |  |  |  |  |  |  |
| DR | 3.854 | 3.755 | 2.629 | 2.581 | 2.335 | 2.761 |  |  |  |  |  |  |
| SG | 4.096 | 3.631 | 2.387 | 2.409 | 2.448 | 3.763 | 1.599 |  |  |  |  |  |
| ZB | 1.645 | 3.784 | 2.523 | 1.571 | 2.342 | 2.381 | 3.175 | 3.476 |  |  |  |  |
| GE1 | 1.871 | 3.560 | 1.767 | 2.025 | 1.914 | 1.554 | 2.137 | 2.584 | 1.712 |  |  |  |
| GE2 | 3.075 | 3.398 | 1.713 | 2.167 | 2.116 | 2.157 | 1.774 | 2.404 | 2.331 | 0.953 |  |  |
| GJ | 2.032 | 3.696 | 1.943 | 2.183 | 2.153 | 1.547 | 1.840 | 2.797 | 1.830 | 0.521 | 1.245 |  |
| CM | 3.729 | 4.533 | 2.510 | 2.662 | 3.086 | 3.985 | 3.369 | 3.413 | 3.727 | 3.089 | 3.116 | 3.264 |

**Table S2.** Pairwise population-based genetic distance (*F_ST_*) between 13 *Ranunculus subrigidus* populations

|  | DT | DL | QM | MQ | MD | NM | DR | SG | ZB | GE1 | GE2 | GJ | CM |
| --- | --- | --- | --- | --- | --- | --- | --- | --- | --- | --- | --- | --- | --- |
| DT | 0.000 |  |  |  |  |  |  |  |  |  |  |  |  |
| DL | 0.751 | 0.000 |  |  |  |  |  |  |  |  |  |  |  |
| QM | 0.685 | 0.619 | 0.000 |  |  |  |  |  |  |  |  |  |  |
| MQ | 0.526 | 0.663 | 0.524 | 0.000 |  |  |  |  |  |  |  |  |  |
| MD | 0.499 | 0.471 | 0.415 | 0.350 | 0.000 |  |  |  |  |  |  |  |  |
| NM | 0.399 | 0.590 | 0.508 | 0.471 | 0.427 | 0.000 |  |  |  |  |  |  |  |
| DR | 0.763 | 0.747 | 0.682 | 0.633 | 0.477 | 0.484 | 0.000 |  |  |  |  |  |  |
| SG | 0.719 | 0.668 | 0.579 | 0.544 | 0.419 | 0.531 | 0.475 | 0.000 |  |  |  |  |  |
| ZB | 0.451 | 0.670 | 0.586 | 0.408 | 0.438 | 0.395 | 0.665 | 0.635 | 0.000 |  |  |  |  |
| GE1 | 0.612 | 0.749 | 0.596 | 0.584 | 0.440 | 0.303 | 0.659 | 0.651 | 0.523 | 0.000 |  |  |  |
| GE2 | 0.664 | 0.671 | 0.503 | 0.537 | 0.402 | 0.353 | 0.540 | 0.547 | 0.542 | 0.375 | 0.000 |  |  |
| GJ | 0.541 | 0.688 | 0.534 | 0.535 | 0.409 | 0.211 | 0.541 | 0.592 | 0.462 | 0.175 | 0.349 | 0.000 |  |
| CM | 0.554 | 0.599 | 0.422 | 0.441 | 0.430 | 0.498 | 0.552 | 0.495 | 0.557 | 0.556 | 0.494 | 0.515 | 0.000 |

**Table S3.** Estimates of historical asymmetric migration rates (*M*)/ number of migrants per generation (*N*m) between populations of *Ranunculus subrigidus*. 1 = DT, 2 = DL, 3 = QM, 4 = MQ, 5 = MD, 6 = NM, 7 = DR, 8 = SG, 9 = ZB, 10 = GE1, 11 = GE2, 12 = GJ, 13 = CM.

|  | ->1 | ->2 | ->3 | ->4 | ->5 | ->6 | ->7 | ->8 | ->9 | ->10 | ->11 | ->12 | ->13 |
| --- | --- | --- | --- | --- | --- | --- | --- | --- | --- | --- | --- | --- | --- |
| 1-> |  | 1.095/1.205 | 1.916/7.648 | 3.945/4.324 | 1.935/6.563 | 3.457/3.175 | 1.489/1.698 | 1.981/2.169 | 1.315/1.044 | 1.795/1.47 | 1.582/2.221 | 2.029/2.22 | 1.476/1.86 |
| 2-> | 1.149/1.484 |  | 2.769/11.052 | 2.64/2.894 | 0.653/2.215 | 0.678/0.623 | 2.903/3.31 | 3.018/3.304 | 1.561/1.24 | 1.183/0.969 | 1.001/1.405 | 2.842/3.11 | 1.986/2.502 |
| 3-> | 1.4/1.808 | 2.716/2.988 |  | 2.141/2.347 | 5.446/18.473 | 2.215/2.035 | 3.663/4.177 | 11.357/12.434 | 3.441/2.733 | 1.452/1.189 | 3.338/4.687 | 4.974/5.443 | 2.124/2.676 |
| 4-> | 4.108/5.305 | 2.863/3.149 | 6.39/25.506 |  | 5.837/19.799 | 3.248/2.983 | 2.312/2.637 | 2.578/2.823 | 2.479/1.969 | 3.723/3.049 | 4.003/5.62 | 2.911/3.185 | 1.78/2.243 |
| 5-> | 2.848/3.678 | 2.285/2.514 | 5.365/21.414 | 3.83/4.198 |  | 3.344/3.072 | 1.875/2.138 | 2.494/2.731 | 3.789/3.01 | 5.956/4.878 | 3.535/4.963 | 3.497/3.827 | 1.892/2.384 |
| 6-> | 3.146/4.063 | 1.91/2.101 | 4.663/18.612 | 3.323/3.642 | 2.378/8.066 |  | 2.611/2.978 | 3.251/3.559 | 2.511/1.994 | 2.151/1.762 | 2.87/4.029 | 2.314/2.532 | 3.095/3.9 |
| 7-> | 2.11/2.725 | 2.616/2.878 | 2.663/10.629 | 2.333/2.557 | 2.478/8.405 | 1.807/1.66 |  | 8.582/9.396 | 4.575/3.634 | 2.062/1.689 | 1.66/2.331 | 7.154/7.828 | 1.837/2.315 |
| 8-> | 1.131/1.461 | 1.899/2.089 | 0.858/3.425 | 1.389/1.522 | 2.494/8.46 | 2.584/2.373 | 7.387/8.424 |  | 2.741/2.177 | 2.225/1.822 | 3.647/5.12 | 0.65/0.711 | 1.478/1.862 |
| 9-> | 1.711/2.21 | 3.421/3.763 | 3.192/12.741 | 9.601/10.524 | 1.713/5.81 | 1.614/1.482 | 1.855/2.115 | 6.343/6.945 |  | 1.798/1.473 | 2.597/3.646 | 2.754/3.014 | 1.484/1.87 |
| 10-> | 2.237/2.889 | 1.18/1.298 | 3.361/13.415 | 3.885/4.258 | 6.032/20.46 | 4.603/4.228 | 1.154/1.316 | 3.712/4.064 | 4.563/3.624 |  | 2.863/4.02 | 4.083/4.468 | 2.468/3.11 |
| 11-> | 2.914/3.763 | 1.084/1.192 | 1.349/5.385 | 2.104/2.306 | 3.906/13.249 | 4.208/3.865 | 1.742/1.987 | 1.385/1.516 | 4.646/3.69 | 7.044/5.77 |  | 3.805/4.164 | 3.787/4.771 |
| 12-> | 2.565/3.313 | 1.432/1.575 | 4.873/19.451 | 1.383/1.516 | 7.177/24.344 | 4.964/4.56 | 2.979/3.397 | 4.525/4.954 | 12.279/9.753 | 4.5/3.686 | 4.753/6.673 |  | 3.308/4.168 |
| 13-> | 1.879/2.427 | 2.256/2.482 | 2.16/8.622 | 2.604/2.854 | 2.608/8.846 | 0.958/0.88 | 2.501/2.852 | 8.195/8.972 | 3.599/2.859 | 2.284/1.871 | 4.845/6.802 |  |  |

**Table S4.** Standardized measures of environmental variables of 13 *Ranunculus subrigidus* populations

| Population | pH | Salinity | GST | ELT | TS | AP |
| --- | --- | --- | --- | --- | --- | --- |
| DT | -0.840 | -0.661 | -0.882 | -1.283 | 0.225 | 1.572 |
| DL | -1.341 | 1.097 | 2.343 | 0.879 | 0.713 | -1.471 |
| QM | -0.913 | 0.950 | -0.807 | -0.974 | 0.385 | -0.322 |
| MQ | 0.192 | -0.856 | -0.589 | -0.974 | 0.202 | 2.024 |
| MD | 0.398 | 1.145 | -0.367 | -0.974 | 0.355 | 0.423 |
| NM | -0.928 | -0.710 | 1.243 | 1.806 | 0.334 | 0.130 |
| DR | -0.766 | -0.759 | 0.292 | 1.188 | 0.416 | 0.121 |
| SG | -0.516 | -0.514 | 0.000 | 1.188 | 0.424 | -0.369 |
| ZB | 0.074 | -0.905 | -1.026 | -0.048 | 0.403 | 0.460 |
| GE1 | 1.061 | 2.171 | 0.589 | -0.048 | 0.330 | -0.143 |
| GE2 | 1.090 | -0.710 | 0.445 | -0.357 | 0.334 | -1.443 |
| GJ | 0.427 | -0.173 | -0.363 | -0.666 | 0.254 | -0.586 |
| CM | 2.062 | -0.075 | -0.881 | 0.261 | 0.575 | -0.397 |

GST: growing season temperature; ELT: extreme low temperate; TS: temperature seasonality; AP: annual precipitation

**Table S5.** Results of dbRDA on the contributions of environmental principal component variables on the genetic pattern. *R*^2^ for each component and the residuals of the model (genetic matrix ~ PC1 * PC2) were represented

|  | PC1 | PC2 | PC1:PC2 | residuals |
| --- | --- | --- | --- | --- |
| Rousset’s *â* | 0.153 ** | 0.111 | 0.067 | 0.669 |
| *F_ST_* | 0.147 * | 0.109 | 0.076 | 0.668 |
| *M* | 0.320 ** | 0.248* | 0.018 | 0.413 |

Significance codes: ‘***’*p*<0.001, ‘**’ 0.001<*p*<0.01, ‘*’ 0.01<*p*<0.05
